# A Comparative Analysis of the Influence of Human Salivary Enzymes on Odorant Concentration in Three Palm Wines

**DOI:** 10.3390/molecules181011809

**Published:** 2013-09-25

**Authors:** Ola Lasekan

**Affiliations:** Department of Food Technology, University Putra Malaysia, UPM 43400, Serdang, Malaysia; E-Mail: lasekan@food.upm.edu.my; Tel.: +603-8946-8535; Fax: +603-8942-3552

**Keywords:** odorants, palm wines, human saliva, aroma extract dilution analysis, stable isotope dilution assays

## Abstract

The influence of human salivary enzymes on palm wines’ odorant concentrations were investigated by the application of aroma extracts dilution analysis (AEDA) and by the calculation of odour activity values (OAVs), respectively. The odorants were quantified by means of stable isotope dilution assays (SIDA), and the degradation profiles of odorants by human saliva were also studied. Results revealed 46 odour-active compounds in the flavour dilution (FD) factor range of 4-256, and all were subsequently identified. Of the 46 odorants, 41 were identified in the *Elaeis guineensis* wine, 36 in *Raphia hookeri* wine and 29 in *Borassus flabellifer* wine. Among the odorants, the highest FD-factors were obtained from acetoin, 2-acetyl-1-pyrroline and 3-isobutyl-2-methoxypyrazine. Among the 13 potent odorants identified, five aroma compounds are reported here as important contributors to palm wine aroma, namely 3-isobutyl-2-methoxy-pyrazine, acetoin, 2-acetyl-1-pyrroline, 3-methylbutylacetate and ethyl hexanoate. Meanwhile, salivary enzymic degradation of odorants was more pronounced among the aldehydes, esters and thiols.

## 1. Introduction

Wine aroma lingers for a considerable time after consumption. Precise sensory evaluation of aroma persistence is rare, mainly due to the fact that determination of perception duration and the exact end point poses some difficulties. Wine aroma perception is a complex sensation triggered when volatile compounds are transported to the olfactory epithelium during wine tasting and consumption. The perception of a volatile depends on the concentration of the compound, threshold levels of the individual, and the duration of exposure [1]. An individual’s perception of a particular wine depends on the amounts and rates of volatile release from the wine matrix. However, the overall perception could also depend on cognitive factors, such as pattern recognition and mood [1]. Wine aroma perception is also influenced by the presence of non-volatile constituents such as polyphenols, polysaccharides and proteins [2,3]. The non-volatile components may interact with aroma compounds in such a way as to affect their volatility and/or concentration in the headspace. This interaction ultimately modifies aroma perception. The impact of these interactions may be concentration-dependent [3]. Ethanol has also been shown to decrease the partition coefficient of various classes of volatile compounds by increasing the solubility of volatile compounds in model wine system [4].

Palm sap is transparent, with a sugar content of 100–144 g/L, a pH of 7.0–7.4 and traces of ethanol. Palm wine, the fermented sap, is whitish and has a pH of about 3.6 and typical alcohol contents of 3.3%–4.0% depending on the stage of fermentation [5]. Like most liquid foods, palm wine is consumed almost immediately (typically within 2–3 s of ingestion); a proportion of the flavour-enriched liquid remains in the mouth as a thin film coating the oral cavity. The thickness of this coating and hence the quality of flavour residing in the mouth, will depend on the viscosity of the film [6].

Perception of wine odorants can, generally, be divided into different stages; first are the ortho-nasal sensations, occurring when the headspace over the wine is sniffed for the highly volatile attributes. Second are the retro-nasally-perceived impressions [7]. At this stage, three key modes have to be distinguished: (a) the immediate aroma impression when wine is present in the oral cavity or (b) has just been swallowed, and (c) the prolonged retro-nasal aroma perception after swallowing, often called “after-taste” or “after-odor” [7]. A detailed explanation of the physiological features influencing aroma transfer from the oral to the nasal cavities has been reported [8]. It was shown that, once food is introduced into the oral cavity, no continuous aroma release into the nasal cavity is possible and distinct physiological actions such as swallowing are necessary to allow or increase aroma perception. The reason for this phenomenon is that the nasal cavity is closed off from the pharyngeal and oral parts by the velum, either forming a tight velum-tongue connection (swallow preparatory phase) or a velopharyngeal closure (pharyngeal phase of swallowing).

While some food aromas can be perceived for just a short period, palm wine aroma lingers for considerable time after consumption [5]. The precise sensory evaluation of aroma persistence is rare, because determination of perception duration and the exact end point poses some difficulties [9]. Recent studies [10,11] have shown that consumption of espresso coffee and palm wine can elicit aroma sensations up to 30 min after consumption, whereas the aroma of Chardonnay wines lasted for only a few minutes. Intensity and duration of prolonged retro-nasal aroma perception, the olfactory components of the so-called after-taste, are of major interest in the food industry.

Buettner *et al.* [12] reported that prolonged retro-nasal aroma perception is highly influenced by physiological factors, such as the adsorption of odorants to the oral mucosa, or salivary constituents effecting the odorants release or retardation in the mouth. The influence of salivary enzymes on food polymer degradation, leading to odorant release from inclusion complexes [13] and odorant metabolisation [10,12] has been reported.

Generally, saliva is a complex mixture containing, not only numerous inorganic constituents, but also a broad variety of organic secretions, such as mucins and a diversity of enzymes [14,15]. The ability of these enzymes to degrade some selected odorous esters, thiols and aldehydes was reported earlier [10]. The influence of saliva macromolecules, such as proteins on the volatility of several odorants has been documented [16,17]. Saliva affects odorant concentration by means of chemical and biochemical reactions between its components and food volatiles. Evidence has been found for the partial hydrolysis of several odor-active acetates [18], as well as ethylic esters, according to their chemical structures. It has been reported that some compounds such as benzaldehyde, diacetyl, ethyl hexanoate and heptyl acetate are affected by the interaction between mucin and the type of solute present [19]. Mucins are high molecular mass glycoproteins responsible for the typical viscosity and elasticity of saliva. They have binding sites, preferentially occupied by sucrose and these sites are also available to trap volatiles [19]. In fact, mucin can bind specific aroma compounds, principally aldehydes [19,20], to form Schiff bases. While our previous study [5] elucidates the influence of human saliva on wine from *E. guineensis*, there are no similar studies on other wines obtained from other palm trees. The present study is aimed at correlating and comparing the effect of human salivary enzymes on key odorants of wines from three different palm trees (*Elaeis guineensis*, *Raphia hookeri*, and *Borassus flabellifer*).

## 2. Results and Discussion

### 2.1. Potent Wine Odorants

Table 1 shows the results of odour qualities and the retention indices of solvent-extracted palm wines and palm wines incubated with saliva (pH 7.8–8.0, 10 min). A total of 46 odorants of which 41 were identified in the oil palm wine (*Elaeis guineensis*, EW) and another 36 in raphia wine (*Raphia hookeri*, RW). On the other hand, the *Borassus flabellifer* wine (BW) yielded only 29 odorants. With the exception of very few odorants, the aroma profiles of EW and RW were quite similar. However, the aroma profile of Borassus wine (BW) showed distinct differences to those of EW and RW respectively. The numbers of odorants identified in wines incubated with saliva varied slightly. While a total of 30 odorants were identified in EW, RW and BW yielded 27 and 26 odorants, respectively.

Generally, most of the odorants were detectable in both fresh wines and wines incubated with saliva. Only ethyl acetate, 2/3-methylbutanal, ethyl lactate, hexyl-3-methylbutanoate, 3-methylthiol-1,1-propanal, 3-mercapto-2-methylpentanone, 4-hydroxy-2,5-dimethyl-3(2*H*)-furanone, ethyl cinnamate, diethyl succinate and γ-dodecalactone were not perceived in the wines incubated with saliva. Moreover, the aroma extracts dilution analysis (AEDA) screening results revealed 31 of the 46 odorants to fall within a flavour dilution (FD)-factor range of 16–256. The compounds with very high FD-factor of at least 64 and above were quantified by stable isotope dilution assays (SIDA) and their odour activity values (OAVs) were calculated (Table 2). In the FD-factor range of 64–256, 13 potent odorants were identified in fresh wines and wines incubated with saliva. The odorants identified include five alcohols, two esters, four heterocyclic compounds of six member rings and two acids. The OAVs revealed that the earthy-smelling 3-isobutyl-2-methoxypyrazine, acetoin and, to a lesser extent, popcorn-like 2-acetylpyrroline and fruity 3-methylbutylacetate and ethyl hexanoate contributed intensely to the fruity, moody aroma nuances of the palm wines.

**Table 1:** Most odour-active volatiles in solvent-extracted palm wines and wines with adjusted pH + saliva.

Compound ^a^	Odour-quality ^b^	Retention FFAP	Index SE-54	FD factor	RW	EW	BW	RWs	EWs	BWs	Previously identified in palm wine ^c^
Acetaldehyde	Pungent fruity	nd	500	4	-	-	+	-	-	+	
Ethyl acetate	Fruity	889	624	8	-	-	+	-	-	-	
2-Methyl butanal	Malty	912	663	16	+	+	-	-	-	-	
3-Methyl butanal	Malty	927	652	16	+	+	+	-	-	-	
Methyl butanoate	Sweet fruity	981	723	16	-	+	-	-	+	-	
2,3-Butandione	Buttery	993	592	16	+	+	+	+	+	+	[21,22]
Ethyl-2-methylbutanoate	Fruity	1040	852	16	+	+	+	+	+	+	
Ethyl pentanoate	Sweet fruity	1067	900	16	-	+	-	-	+	-	[22]
2-Heptanone	Soapy fruity	1181	891	8	-	-	+	-	-	+	
2/3-Methylbutanol	Malty	1213	738	64	+	+	+	+	+	+	
Ethyl hexanoate	Fruity	1226	1001	64	+	+	+	+	+	+	[21]
Acetoin	Buttery	1275	nd	256	+	+	+	+	+	+	
Ethyl lactate	Phenolic/smoky	1321	nd	8	+	+	-	-	-	-	
2-Acetyl-1-pyrroline ^d^	Popcorn	1323	922	256	+	+	+	+	+	+	
3-Hydroxy-2-butanone	Buttery	nd	718	8	-	-	+	-	-	+	
1-Hexanol	Rubbery	1356	872	4	+	+	+	+	+	+	
Acetic acid	Sweaty	1428	600	16	+	+	+	+	+	+	[21,22]
Ethyl octanoate	Fruity	1429	1199	8	+	-	-	+	-	-	
Hexyl-3-methyl butanoate	Fruity	1430	nd	16	+	+	-	-	-	-	
2-Ethyl 3,5-dimethylpyrazine	Roasty	1451	1083	64	+	+	-	+	+	+	
Methional	Cooked potato	1460	nd	8	+	+	+	+	+	+	[22]
3-Isobutyl-2-methoxypyrazine	Earthy	1517	1175	256	+	+	+	+	+	+	
3-Methylbutyl acetate	Banana	1527	878	128	+	+	-	+	+	-	
2-Acetylpyridine	popcorn	1532	nd	64	+	+	+	+	+	+	
Linalool	Fresh-blooming	1540	1103	64	+	+	+	+	+	+	
2-Methylpropanoic acid	Sweaty	1563	nd	128	+	+	+	+	+	+	
Butanoic acid	Sweaty-buttery	1619	821	8	+	+	-	+	+	-	
2/3-Methylbutanoic acid	Sweaty	1661	875	16	+	+	+	+	+	+	
(z)-1-5-Octadien-3-one	Geranium-like	1676	984	4	+	+	+	+	+	+	
( *E,E*)-2,4-Nonadienal	Fatty	1718	1215	8	-	+	-	-	+	-	
Pentanoic acid	Sweaty	1720	911	16	+	+	+	+	+	+	
3-Methylthiol-1,1-propanal	Broth-like	1723	903	8	+	+	-	-	-	-	
3-Mercapto-2-methylpentanone^ d^	Gravy-meaty	1742	883	16	-	+	-	-	-	-	
β-Damascenone	Flowery	1801	1389	16	+	+	+	+	+	+	
2-Methoxyphenol	Smoky	1842	1089	64	+	+	+	+	+	+	
2-Phenylethanol	Honey	1911	1117	128	+	+	+	+	+	+	
4-Hydroxy-2,5-dimethyl-3(2H)-furanone	Caramel-like	2038	1070	16	+	+	+	-	-	-	
3-Methylpentanoic acid	Sweaty	2040	nd	8	+	+	+	+	+	+	
Homofuraneol	Apple-like	2095	nd	16	+	+	-	+	+	-	
Ethyl cinnamate	Sweet	2167	1469	8	-	+	-	-	-	-	
Sotolone	Spicy	2190	1110	8	+	+	+	+	+	+	
Diethyl succinate	Sweet-pineapple	2390	nd	4	+	+	-	-	-	-	
y-Dodecalactone	Fruity	2424	1497	16	+	+	+	-	-	-	
Phenylacetic acid	Honey	2577	1262	64	+	+	+	+	+	+	
Vanillin	Vanilla	2601	1404	16	+	+	-	-	-	-	
4-Methoxymethylphenol	Phenolic	2639	nd	8	-	+	-	-	+	-	

^+^ Presence of compound in wine and ^−^ Absence of compound in wine; ^a^ Compounds were identified by comparing them with reference substances on the basis of the following criteria: retention index (RI) on different stationary phases given in the table, mass spectra obtained by MS (EI) and MS (CI), and odour quality as well as odour intensity perceived at the sniffing port. ^b^ Odour quality perceived at the sniffing port. ^c^ Reported in the literature as volatile compounds of palm wine: Jirovetz *et al.* (2001) [21]; Uzochukwu *et al.* (1994) [22]; ^d^ The MS signals were too weak for an unequivocal interpretation. The compounds were identified on the basis of the remaining criteria given in foot note -a. ^RWS^ *Raphia hookeri* wine + saliva; ^EWS^ Palm wine (*E. guineensis)* + saliva; ^BWS^ *Borassus flbellifer* wine + saliva.

**Table 2:** Concentration of potent odorants in wines (RW, EW and BW) and wines + saliva (µg/L) ^a^.

No	Compounds	RW	EW	BW	RWS	EWS	BWS	OAVs (RW) ^b^	OAVs (EW) ^b^	OAVs (BW) ^b^	OTW (µg/L) ^c^
1	3-Methyl butanol	19127 ± 25 ^a^	18300 ± 34 ^b^	18109 ± 15 ^c^	19315 ± 12 ^a^	18360 ± 19 ^b^	18315 ± 15 ^b^	19.1 ^a^	18.3 ^b^	19.3 ^a^	1000
2	Ethyl hexanoate	61.9 ± 3.2 ^a^	52.2 ± 7.1 ^b^	48.4 ± 5.2 ^c^	53.2 ± 6.3 ^a^	41.5 ± 3.8 ^b^	39.7 ± 4.6 ^c^	61.9 ^a^	52.2 ^b^	48.4 ^c^	1
3	Acetoin	712100 ± 21 ^a^	663500 ± 15 ^b^	452120 ± 10 ^c^	527100 ± 25 ^a^	410500 ± 14 ^b^	235600 ± 12 ^c^	890.13 ^a^	829.4 ^b^	565.15 ^c^	800
4	2-Acetylpyrroline	9.8 ± 0.2 ^b^	11.4 ± 0.1 ^a^	5.3 ± 0.1 ^c^	9.6 ± 0.1 ^b^	11.3 ± 0.2 ^a^	5.0 ± 0.1 ^c^	98 ^b^	114 ^a^	53^c^	0.1
5	2-Acetylpyridin	0.32 ± 0.0 ^b^	0.32 ± 0.0 ^b^	0.45 ± 0.0 ^a^	0.30 ± 0.0 ^b^	0.30 ± 0.0 ^b^	0.41 ± 0.0 ^a^	nd	nd	nd	nd
6	2-Ethyl-3,5-dimethylpyrazine	0.25 ± 0.0 ^b^	0.47 ± 0.0 ^a^	nd	0.23 ± 0.0 ^b^	0.46 ± 0.0 ^a^	nd	1.56 ^b^	2.9 ^a^	nd	0.16
7	3-Isobutyl-2-methoxypyrazine	10.9 ± 0.0 ^b^	12.0 ± 0.0 ^a^	9.7 ± 0.0 ^c^	11.2 ± 0.0 ^b^	12.5 ± 0.1 ^a^	9.9 ± 0.0 ^c^	2180 ^b^	2400 ^a^	1940 ^c^	0.005
8	3-Methylbutyl acetate	70.12 ± 5.2 ^a^	61.72 ± 3.1 ^b^	nd	68.50 ± 4.3 ^a^	59.73 ± 3.0 ^b^	nd	79.7 ^a^	70.0 ^b^	nd	0.88
9	Linalool	8.74 ± 0.1 ^c^	11.22 ± 0.1 ^b^	13.60 ± 0.1 ^a^	8.72 ± 0.1 ^c^	11.0 ± 0.1 ^b^	13.30 ± 1.2 ^a^	1.46^c^	1.9 ^b^	2.3 ^a^	6
10	2-Methylpropanoic acid	1650 ± 11 ^b^	1680 ± 9.0 ^b^	1735 ± 10 ^a^	1560 ± 8.5 ^b^	1580 ± 5.0 ^b^	1640 ± 9.0 ^a^	<1	<1	<1	8100
11	2-Methoxy phenol	0.34 ± 0.0 ^b^	0.28 ± 0.0 ^c^	0.40 ± 0.0 ^a^	0.34 ± 0.0 ^b^	0.28 ± 0.0 ^c^	0.40 ± 0.0 ^a^	<1	<1	<1	3
12	2-Phenyl ethanol	6570 ± 10 ^a^	5880 ± 8.0 ^b^	4870 ± 10 ^c^	6120 ± 15 ^a^	5470 ± 8.2 ^b^	4380 ± 10 ^c^	6.57 ^a^	5.88 ^b^	4.87 ^c^	1000
13	Phenylacetic acid	365 ± 4.5 ^c ^	417 ± 7.0 ^b^	520 ± 5.0 ^a^	350 ± 6.0 ^c^	402 ± 4.1 ^b^	506 ± 6.0 ^a^	<1	<1	<1	10000

^a^ Data are mean values of triplicate determination, data with different superscript within the same roll are significantly (*p* < 0.05) different. ^b^ OAV, odour activity values were calculated by dividing the concentrations of the odorant by their ortho-nasal odour threshold in water. ^c^ Odour threshold reported in the literature. ^RW^ *Raphia hookeri* wine; ^EW^ Palm wine (*E. guineensis*); ^BW^ *Borassus flbellifer* wine. ^RWS^ *Raphia hookeri* wine + saliva; ^EWS^ Palm wine (*E. guineensis)* + saliva; ^BWS^ *Borassus flbellifer* wine + saliva.

The potent odorants with significantly high concentration in the wines were acetoin (4.5 × 10^5^–7.1 × 10^5^ µg/L), 3-methylbutanol (1.8 × 10^4^–1.9 × 10^4^ µg/L), 2-phenyethanol (4.87 × 10^3^–6.57 × 10^3^ µg/L), 2-methylpropanoic acid (1.61 × 10^3^–1.73 ×10^3^ µg/L) and phenylacetic acid (Table 2). Significant (*p* < 0.05) differences were noticed in the concentration of the potent odorants isolated from the different wines.

### 2.2. Influence of Saliva on the Palm Wine Odorants

#### 2.2.1. Pyrazines and Alcohols

After 1, 5, and 10 min of incubation with saliva at pH 7.5–8.0, no noticeable degradation was obtained among the pyrazines and pyrrolines, namely, 3-isobutyl-2-methoxypyrazine, 2-ethyl-3,5-dimethylpyrazine, 2-acetylpyrroline and 2-acetylpyridine (Table 2). Similar observations were recorded for the alcohols, such as methoxyphenol, and linalool. Acetoin was, however, significantly degraded. In the case of 3-methylbutanol and 2-phenylethanol, noticeable but insignificant degradation occurred. For instance, 2-phenylethanol suffered approximately 7% degradation after incubation with saliva for 10 min. On the other hand, 3-methylbutanol was increased by <1%. This observation is in agreement with those of Buettner *et al.* [7]. 

#### 2.2.2. Aldehydes and Esters

**Figure 1:**
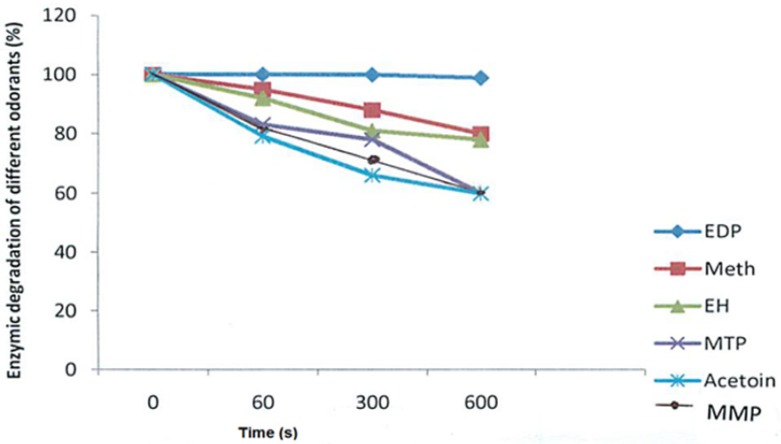
Enzymatic degradation of selected palm wines’ odorants [2-ethyl-3,5-dimethylpyrazine (EDP), methional (Meth), ethyl hexanoate (EH), 3-methylthiol-1-propanal (MTP), 3-mercapto-2-methylpentanone (MMP) and acetoin during incubation with saliva at different time intervals.

**Figure 2:**
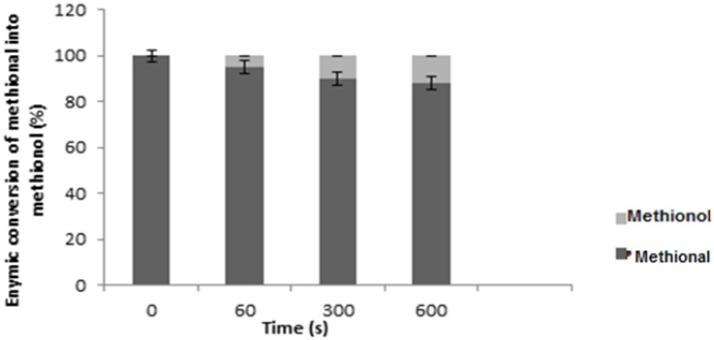
The conversion of palm wine methional to methionol during incubation with saliva at different time intervals [60, 300 and 600 s]. Initial concentration of methional = 100 µg/L. values are the means of three replicates. Error bars show the standard deviations.

In contrast to the pyrazines and alcohols, there were significant degradations in aldehydes and esters incubated with saliva. Figure 1 gives an insight into the influence of saliva on aldehydes, esters and alcohols. Methional was reduced by approximately 19% after 10 min of incubation with saliva. Moreover, the decrease of methional was related to the formation of methionol (Figure 2), indicating that reduction after incubation is the obvious reaction occurring with saliva. 3-Methylbutanal was found to follow the same reaction, being reduced to 3-methylbutanol. The experiment on 3-methylbutanal was repeated three times with three different samples of saliva from one panellist on three different days. It was found that the enzymic degradation of 3-methylbutanal could vary by 10%–18% from one day to the other, indicating that reductive salivary activity for one panellist is not fully consistent. This effect has already been previously observed for the degradation of model homologous aliphatic aldehydes in the presence of saliva [12]. After thermal treatment of the saliva (100 °C, 10 min), no degradation of the aldehydes was observable (data not shown). At 100 °C, other effects could have occurred such as protein denaturation, protein aggregation and precipitation or change of the physicochemical properties of the saliva, in particular viscosity. These events could also explain some of the obtained results. The factors inducing salivary reduction of the investigated aldehydes cannot be confirmed at present. In a previous study on white and red wines, Friel and Taylor [19] reported significant interaction between aldehydes and saliva mucin. They showed that aldehydes can bind to mucin to form Schiff bases. Generally, the two major metabolic pathways for aldehydes in human beings are; oxidation to carboxylic acids and reduction to the corresponding alcohols, with the first being catalysed by NAD-linked alcohol dehydrogenases and by NADP-linked aldehyde reductases [23]. The esters (ethyl hexanoate and 3-methylbutyacetate) were more degraded than the aldehydes. The most probable factor responsible for the degradation of esters is hydrolysis, as many esterolytic enzymes can be found in human saliva [24]. The decrease of esters in wine with human saliva has been attributed to carboxylesterases [14,18]. Nevertheless, the action of mucin cannot be excluded. It’s possible that saliva mucin can also establish hydrophobic bonds with aroma compounds, causing a decrease in concentration as previously demonstrated for ethyl hexanoate and heptyl acetate by Friel and Taylor [19]. The behaviour observed for these two compounds is enhanced by the interaction between mucin and solute salivary components. According to Friel and Taylor [19], salivary salts may modify the number of available binding sites of mucin and may also result in the formation of hydrophobic inclusion sites that can trap volatiles within the solution structure. This could also explain the decrease in the level of esters. Although, the presence or absence of bacteria in the saliva medium was not investigated in this study, previous reports have shown that bacteria in the saliva are capable of hydrolysing/or oxidising different aroma compounds [25].

#### 2.2.3. Thiols

3-Methylthiol-1-propanal (MTP) (Figure 1) and 3-mercapto-2-methylpentanone were greatly degraded. The ability of thiols to function as peroxidase substrates has been described [26,27]. Interestingly, peroxidase activity assays have also been performed by the use of guaiacol an important aroma compound in foods as the substrate [28]. Also, the influences of pH, hydrogen peroxide and thiocyanate on thiols have been investigated [26], the two latter compounds being general constituents of human saliva [27].

### 2.3. Odour Activity Values (OAVs)

To estimate the respective contribution of the odorants to the wines’ aroma profile, the OAVs of the odorants were calculated on their nasal odour thresholds in water (Table 2). The OAVs showed that 3-isobutyl-2-methylpyrazine, acetoin and to lesser extent, 2-acetylpyrroline, 3-methylbutylacetate and ethylhexanoate, contributed intensely to the fruity-moody aroma of the wines. Interestingly, odorant compounds with high concentration in the wines such as 3-methylbutanol, 2-phenylethanol and phenylacetic acid produced relatively low orthonasal OAVs. These compounds contribution to the overall orthonasal aroma quality of the wines would be low. Results also revealed some compounds (ethyl hexanoate, 2-acetylpyrroline and 3-methylbutylacetate) with OAVs higher than their corresponding orthonasal odour threshold in water. While these compounds might not play much important role during sniffing of the wines, they might have significant impact during the consumption of the wines. A series of interaction phenomena, such as additive, synergistic or suppressive effects, are well-documented, so that the presented OAVs do not allow a direct prediction of the odorants’ contribution to the wine aroma sensation.

## 3. Experimental

### 3.1. Materials

#### 3.1.1. Raw Materials

Three bottles (4.5 L) of each wine obtained from three different palm trees (*E. guineensis*, *R. hookeri and B. flabellifer*) were freshly purchased directly from the production farm in a sterilized containers encrusted in ice. The samples were bottled, pasteurized and later dispensed into 45 mL glass-tubes and stored at −20 °C prior to analysis. The alcohol contents of the palm wine samples were 3.7% (*E. guineensis*), 4.0% (*R. hookeri)* and 3.2% (*B. flabellifer*), respectively. 

### 3.2. Chemicals

The following odorants; methyl butanoate, 99%; 2/3-methyl-1-butanol, 98%; ethyl hexanoate, 97%; acetoin, 98%; ethyl lactate, 99%; methional, 99%; 3-isobutyl-2-methoxypyrazine, 70%; 3-methylbutylacetate, 99%; linalool, 98%; butanoic acid, 97%; 2/3-methylbutanoic acid, 98%; 3-methylthiol-1-propanal, 98%; 2-methoxyphenol, 98%; 4-hydroxy-2,5-dimethyl-3(2*H*)-furanone, 98%; 3-methylpentanoic acid, 98%; ethyl cinnamate, 98%; diethyl succinate, 99%; and phenylacetic acid, 99% were purchased from (Aldrich, Steinheim, Germany), while, 2,3-butandione, 99%; 2-acetylpyridine, 99%; and pentanoic acid, 98% were obtained from (Fluka, Neu-Ulm, Germany). Others such as 2-ethyl-3,5-dimethylpyrazine, 98%; 2-phenylethanol, 99%; acetic acid, 99% and vanillin, 98% were purchased from (Acros Organics, Morris Plain, NJ, USA and Merck, Darmstadt, Germany) respectively. The compounds were freshly distilled prior to analysis. Chemical and sensory purity was checked by high resolution gas chromatography-olfactometry (HRGC/O) as well as high resolution gas chromatography-mass spectrometry (HRGC-MS).

### 3.3. Stable-Isotope-Labelled Standards

The following labelled internal standards were synthesized according to the cited literature: [^2^H_3_]-methylbutanol [29]; 2-phenyl [1,1^2^H_3_] ethanol [29]; 2-[^2^H_3_] methoxyphenol [30]; 3 [^2^H_3_] methylthiol-propanal [31]; 3-methyl [3,4-^2^H_2_] butyl acetate [30]; [2,2,2-^2^H_3_] ethyl hexanoate [30]; 3-isobutyl-2[^2^H_3_] methoxypyrazine [32]; [^2^H_3_] vanillin [30]; [^13^C_2_] acetic acid and phenylacetic acid were obtained from (Aldrich). The concentrations of the labelled internal standards and the response factors (FID) were determined gas chromatographically, using methyl octanoate as the internal standard as described by Buettner and Schieberle [33]. The calibration factors for the labelled compounds were calculated as reported by Sen *et al.* [31] (Table 3).

**Table 3:** Selected ions and calibration factors used for quantitation by stable isotope dilution assay (SIDA).

No	Compounds ^a^	Ions ( *m/z*)	Internal standard	Ion ( *m/z*)	Calibration factor ^b^
1	2-Methyl butanol	71	[^2^H_3_]-2-Methyl butanol	74	0.88
2	3-Methyl butanol	71	[^2^H_3_]-2-Methyl butanol	74	0.88
3	Ethyl hexanoate	145	[2,2,2,-^2^H_3_]-Ethyl hexanoate	148	1.0
4	Acetoin	91	^13^C_2_-Acetoin	93	0.89
5	2-Acetylpyrroline	60	[^2^H_3_]-Acetylpyrroline	61	1.0
6	Acetic acid	61	[^13^C_2_]-Acetic acid	63	1.0
7	2-Ethyl 3,5-dimethylpyrazine	137	2-Ethyl [3,5-^2^H_3_] dimethylpyrazine	140	1.0
8	Methional	105	3-([^2^H_3_]-Methylthiol)-1-propanal	108	0.71
9	3-Isobutyl-2-methoxypyrazine	167	3-Isobutyl-2-[^2^H_3_]-methoxypyrazine	170	0.95
10	3-Methylbutyl acetate	131	3-Methyl [3,4-^2^H_3_]-methoxypyrazine	133	0.79
11	2-Acetylpyridine	53	2-[^2^H_3_]-Acetylpyridine	54	1.0
12	Linalool	137	Tetrahydrolinalool	141	1.61
13	2-Methoxyphenol	125	2-[^2^H_3_]-Methoxyphenol	128	1.0
14	2-Phenylethanol	105	2-phenyl-[1,1,-^2^H_2_] ethanol	107	1.0
15	Phenylacetic acid	137	[^13^C_2_]-Phenylacetic acid	139	1.0
16	Vanillin	151	[^2^ H_3_]-Vanillin	156	1.01

^a^ Compounds were determined using the respective stable isotope labelled standards by means of the ion trap detector ITD-800 (Finnigan, Bremen, Germany) running in the C I mode with methionol as reagent gas.

^b^ The calibration factor was determined as reported by Sen *et al.* [31].

### 3.4. Enzymatic Analyses

#### 3.4.1. Collection of Saliva and Enzyme Assay

Mixed whole resting saliva (10 mL) was collected separately from four panellists 2 h after breakfast and after thorough cleaning of the teeth and was used immediately for analysis. Panellists (four males and four females) were volunteers (non-smokers) from the Technical University of Munich, exhibiting no known illnesses at the time of examination and with normal olfactory and gustatory functions. Subjective aroma perception was normal in the past and at the time of examination, before sampling, each panellist rinsed his/her mouth several times with tap water to avoid contamination.

#### 3.4.2. Interaction of Wine with Saliva

Saliva (10 mL) obtained from panellists was immediately used for analysis. Wine (10 mL) was kept in a flask sealed with a lid, and thermostatted at 37 °C after application of 1 mL whole human saliva [25], the solution was stirred at 37 °C for 10 min [11]. The pH of the wine solution containing saliva was always between 7.5 and 8.0. Then, 10 mL of a saturated CaCl_2_ solution was added to inhibit enzymatic processes, and the mixture was immediately subjected to quantitation [33]. Each experiment was performed in triplicate. A reference analysis was performed in parallel by using a sample (10 mL of wine) in exactly the same way but without adding saliva. Furthermore, a blank (10 mL of saliva solution) was run in exactly the same way as was done with the wine samples. Therefore, contamination of samples with odorants originating from saliva was excluded.

#### 3.4.3. Inhibition of Enzymatic Activity

The same experiments were performed after thermal treatment of saliva samples in a closed vessel (100 °C, 10 min). The saliva was cooled to 37 °C and immediately applied for the enzyme assays, as described above.

### 3.5. Quantitation of the Odorants by Stable Isotope Dilution Assays

After the enzyme assay, the solution was immediately spiked with known amounts of the labelled internal standards listed in Table 3, stirred for equilibration (20 min), and extracted with dichloromethane (three times, total volume = 300 mL). The combined organic extracts were dried over anhydrous Na_2_SO_4_, and then concentrated to a total volume of 200 µL [34] and subsequently analysed by multidimensional GC-MS.

### 3.6. Chromatography-Mass Spectrometry

The odorants were quantified by two-dimensional gas chromatography (TD-HRGC), using a Mega 2 gas chromatography (Fisons Instruments, Mainz-Kastel, Germany) as the precolumn system in tandem with a Fisons GC 5160 as the main column system. MS analyses were performed with an ITD-800 (Fisons Instruments) running in the C I mode with methanol as the reagent gas. The following fused silica columns were used: DB- FFAP (30 m × 0.32 mm i.d. 0.25 µm FD, J & W Scientific, Folsom, CA, USA) in combination with DB-5 (SE-54; 30 m × 0.32 mm i.d., 0.25 µm FD, J & W Scientific). The gas chromatographic conditions were as described by Buettner and Schieberle [33]. The concentrated wine extracts were applied by the ‘cool’-on column injection technique at 40 °C. After 2 min, the temperature of the oven was raised at 4 °C/min to 50 °C and held for 2 min isothermally at the same temperature. The oven temperature was later raised at 6 °C/min to 180 °C and finally raised to 230 °C at 15 °C/min. The flow rate of the carrier gas (helium) was 2.5 mL/min. 

### 3.7. Aroma Extracts Dilution Analysis (AEDA)

The FD factors of the odour-active compounds were determined by AEDA [35] using the following dilution series; the original wine extracts (400 µL) from 600 mL of fresh palm wine was specially diluted with diethyl ether (1:1) until no odorant of wine was detectable by sniffing of the highest dilution. HRGC/O was performed with aliquots (0.5 µL), using capillary FFAP. In total, three experienced sniffers were used to perform the AEDA experiments. Only the odours detected by all the three panellists were considered valid. Their response (sensitivity) to individual compounds did not differ by > 2 FD factors.

### 3.8. Identification of Volatile Compounds

Compounds were identified by comparison with the reference substances on the basis of the following criteria: retention index (RI) on two stationary phases of different polarities, mass spectra obtained by MS (EI) and MS (CI), and odour quality, as well as odour intensity perceived at the sniffing port. Odour intensity was checked by GC/O and by comparing the FID signal caused by a defined amount of each reference aroma compound.

### 3.9. Calculation of Odour Activity Values (OAVs)

The OAVs were calculated by dividing the concentrations of the odorants by their ortho-nasal odour threshold in water.

### 3.10. Statistical Treatment of Data

The data obtained were subjected to the analysis of variance (Tukeys test, α = 0.05). Statistical data processing was performed using the Stat graphics plus (5-PC) Statistical package (Manugistics Inc., Rockville, MD, USA, 1999).

## 4. Conclusions

A total of 46 odorants were detected in the tested palm wines, of which 41 were identified in the oil palm wine (*Elaeis guineensis*, EW) and another 36 in raphia wine (*Raphia hookeri*, RW). On the other hand, the *Borassus flabellifer* wine (BW) yielded only 29 odorants. The SIDA results revealed 13 potent odorants in both fresh wines and wines incubated with saliva. Of the 13 potent odorants, the OAVs showed that 3-isobutyl-2-methylpyrazine, acetoin and to lesser extent, 2-acetylpyrroline, 3-methylbutylacetate and ethyl hexanoate, contributed intensely to the fruity-moody aroma of the wines. While enzymic degradation of odorants in the presence of saliva was not noticeable among the pyrazines, pyrrolines and most alcohols, it was, however, quite pronounced among the aldehydes, esters and thiols.
